# Supplementary material for: Protein Posttranslational Signatures Identified in COVID-19 Patient Plasma
Source: Front Cell Dev Biol. 2022 Feb 11;10:807149. doi: 10.3389/fcell.2022.807149 (PMC8873527; doi:10.3389/fcell.2022.807149)
Supplement: Supplementary file 4 [file Presentation1.pdf]

Protein Posttranslational Signatures Identified in COVID-19 Patient Plasma

Pavan Vedula[1], Hsin-Yao Tang[2], The UPenn COVID Processing Unit[3], David W. Speicher [2], and Anna Kashina[1*]

[1]Department of Biomedical Sciences, University of Pennsylvania School of Veterinary Medicine, [2]The Wistar Institute, Philadelphia, 19104, USA

SUPPLEMENTAL ONLINE INFORMATION

Vedula_et_al_Fig.S1

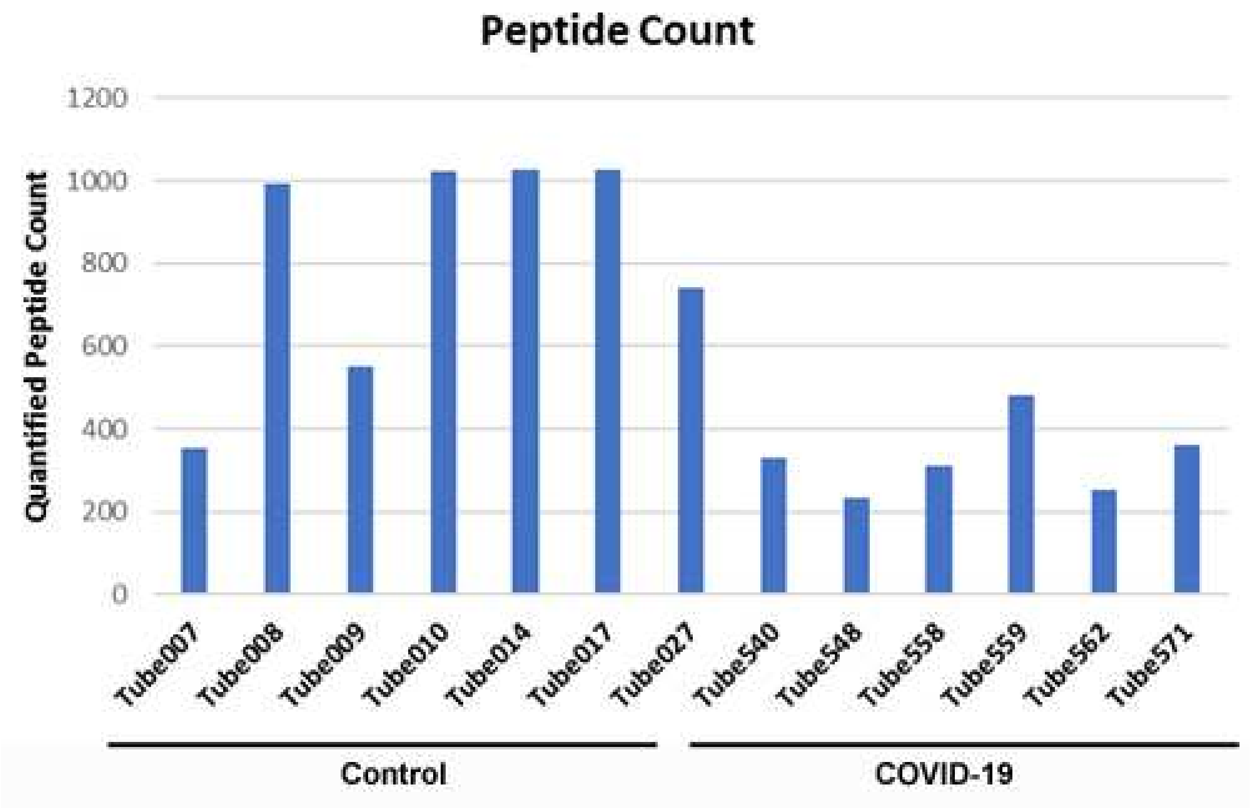

Figure S1. Overall number of plasma peptides identified by peptidomics is lower in COVID-19 compared to control. Numbers of identified peptides from each sample are shown, samples 007-027 are control and 540-571 are COVID-19.

Vedula_et_al_FigS2

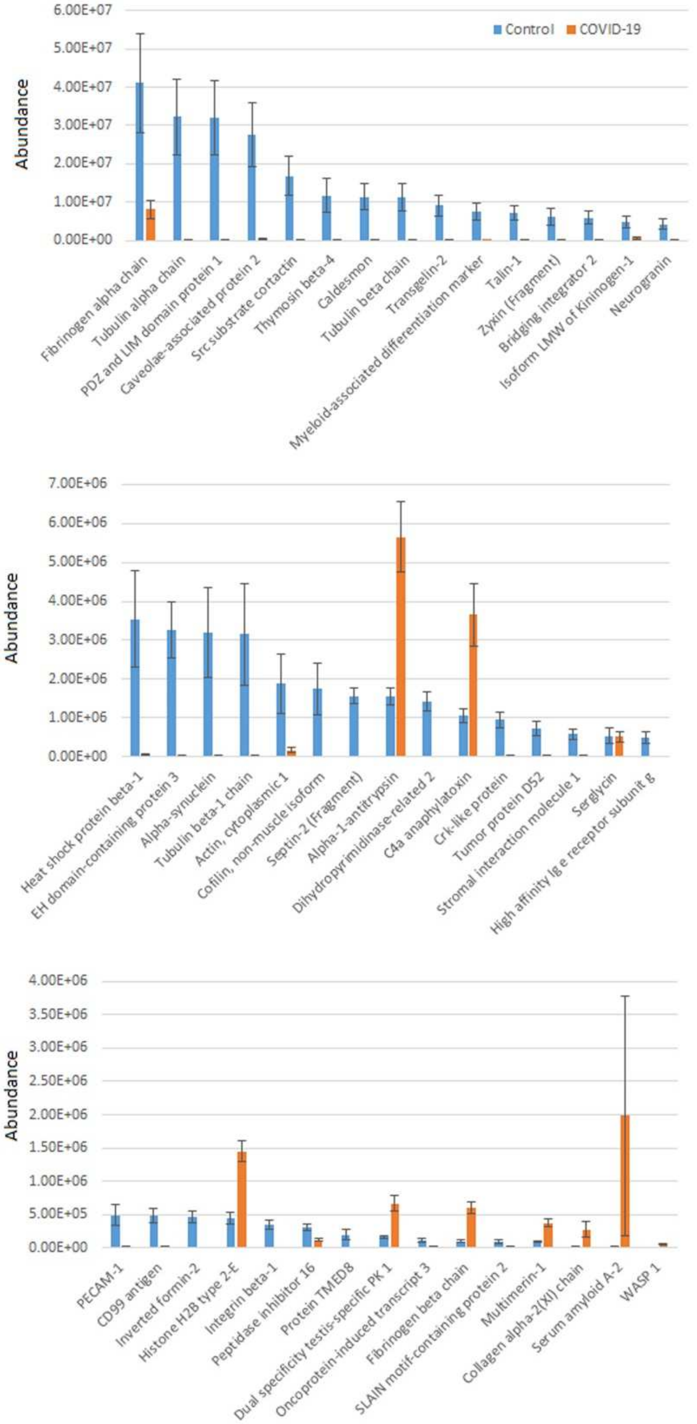

Figure S2. Plasma peptides from COVID-19 patients exhibits prominent changes in composition compared to control. Combined normalized intensities of all significantly changed peptides for each parent protein listed on the X axis. Bars represent normalized intensity levels averaged for all samples in each group. Proteins with lower combined intensities are shown. See Fig. 1 for the highest abundance hits. Error bars represent SEM (n=7 for control, 6 for COVID-19).

Vedula_et_al_Fig.S3

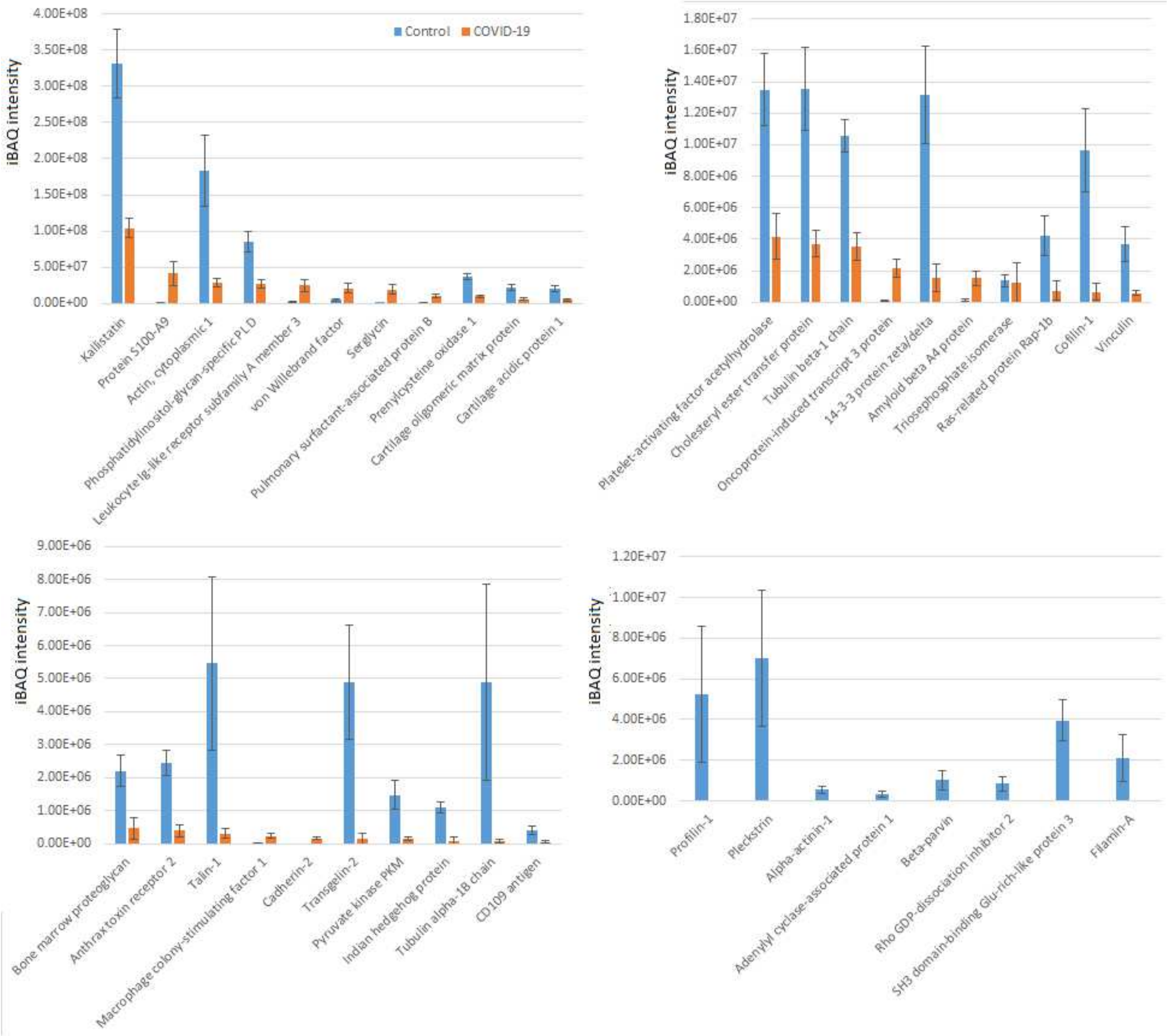

Figure S3. Plasma proteins from COVID-19 patients exhibits prominent changes compared to control. iBAQ Intensities of the less abundant proteins showing significant differences between COVID-19 and control plotted on different scales. See Fig. 3 for the most abundant hits. Bars represent normalized intensity levels averaged for all samples in each group, error bars represent SEM (n=7 for control, 6 for COVID-19).

Vedula_et_al_Fig.S4

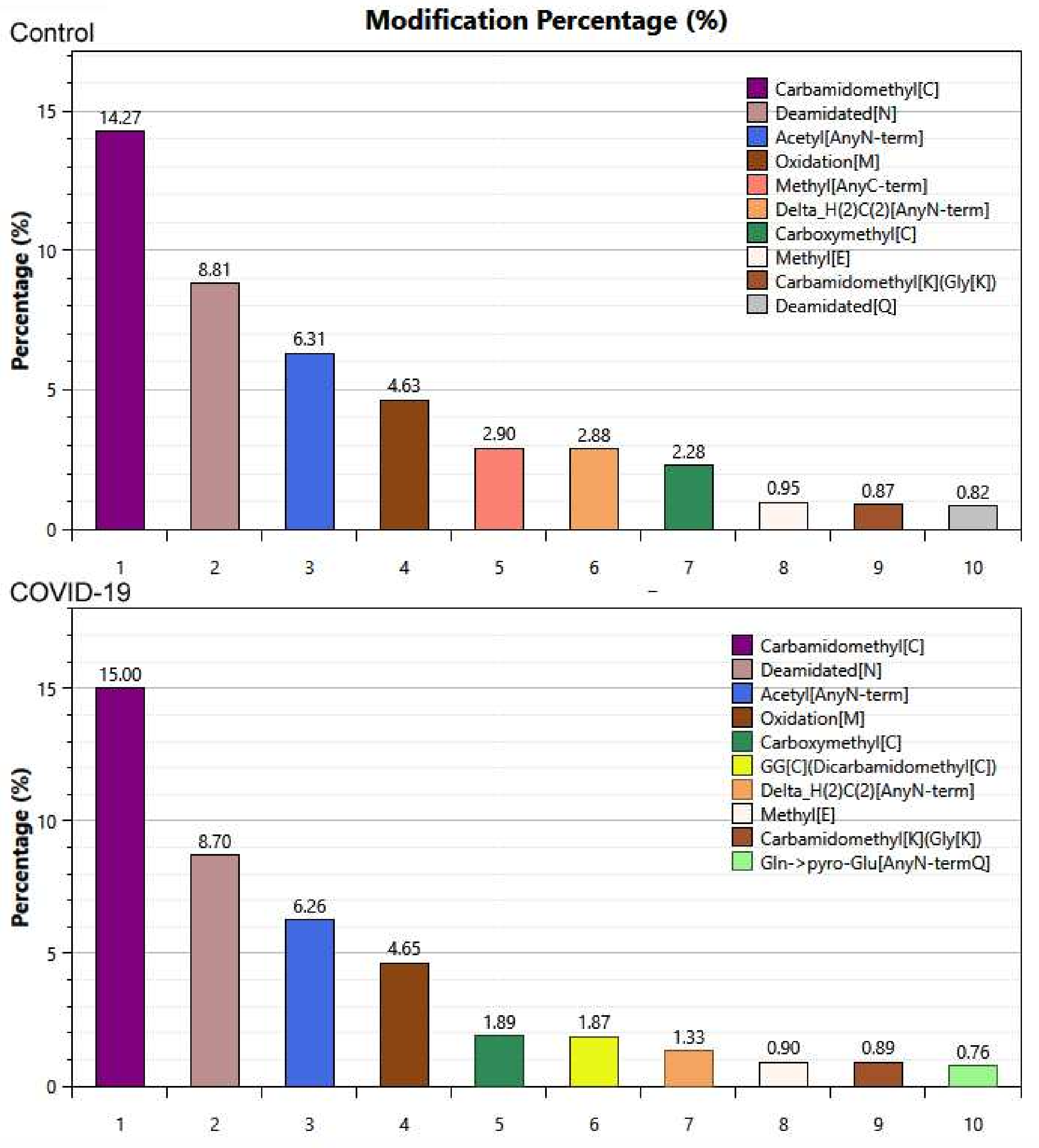

Figure S4. Top abundance posttranslational modifications identified in control and COVID-19 plasma samples. Percentages of modification derived directly from pFind search are shown.

Vedula_et_al_Fig.S5

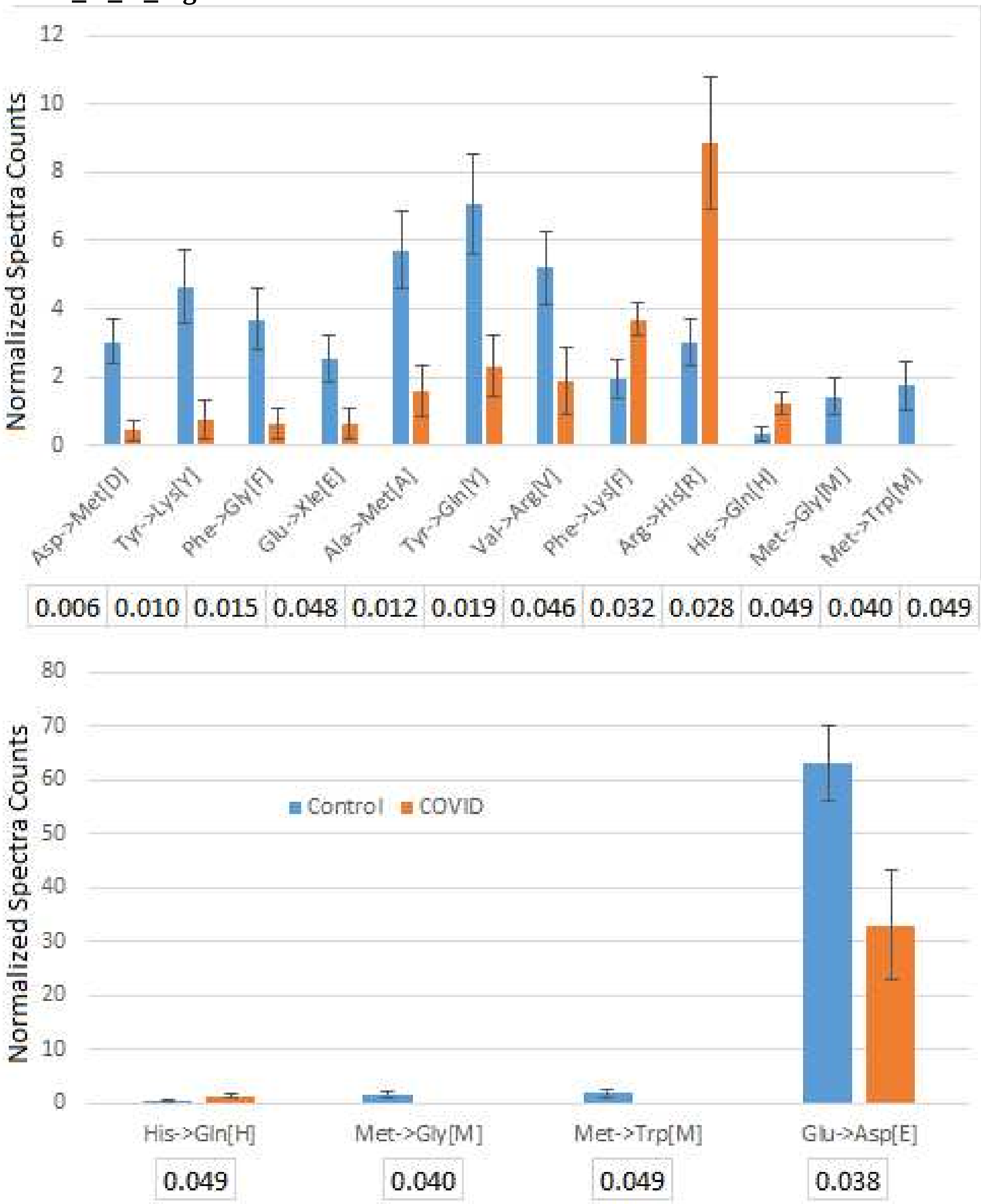

Figure S5. Plasma proteins from COVID-19 patients exhibits altered patterns of amino acid substitutions indicative of single nucleotide polymorphisms. Error bars represent SEM (n=7 for control, 6 for COVID-19). P values were calculated by 2-tailed Student's T-test. P values calculated by 2-tailed Student's T-test are listed underneath each set. Three last sets on the top chart are also duplicated in the bottom chart for scale.

<u>Dataset, Tables and Supplemental Tables:</u>

Dataset 1. Sample spectra from peptides identified as arginylated in our analysis.

Table S1. List of patient samples used in this study.

Table S2. Peptides exhibiting significant differences between control and COVID-19 plasma.

Table S3. Peptides from the peptidome analysis that did not exhibit significant differences between control and COVID-19 plasma.

Table S4. Proteins exhibiting significant differences between control and COVID-19 plasma.

Table S5. Proteins that did not exhibit significant differences between control and COVID-19 plasma.

Table S6. Significant PTMs identified by pFind search used for the chart in this study.

Table S7. Individual and combined results from pFind search listing the modification sites in each sample.

Table S8. Proteins and sites identified as arginylated in control and COVID-19 plasma.

Table S9. Proteins and sites identified as phosphorylated on Thr in control and COVID-19 plasma.
