# Supplementary material for: Protein Posttranslational Signatures Identified in COVID-19 Patient Plasma
Source: Front Cell Dev Biol. 2022 Feb 11;10:807149. doi: 10.3389/fcell.2022.807149 (PMC8873527; doi:10.3389/fcell.2022.807149)

Dataset 1

Selected LC-MS/MS spectra of arginylated peptides found in COVID-19 and control plasma samples

(arginylated spectra are denoted in red in each sequence)

Alpha-1-antitrypsin; D36

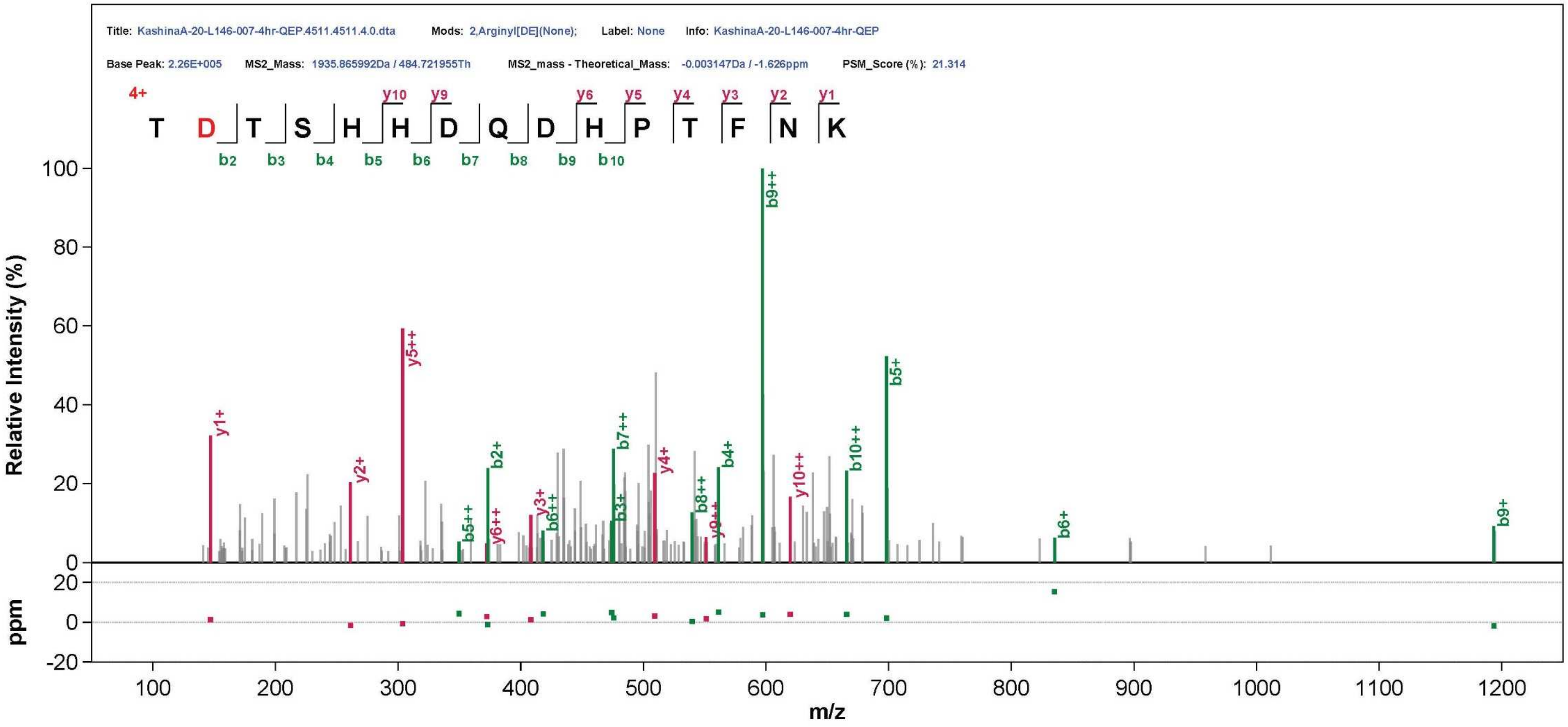

Fibrinogen alpha chain; E30

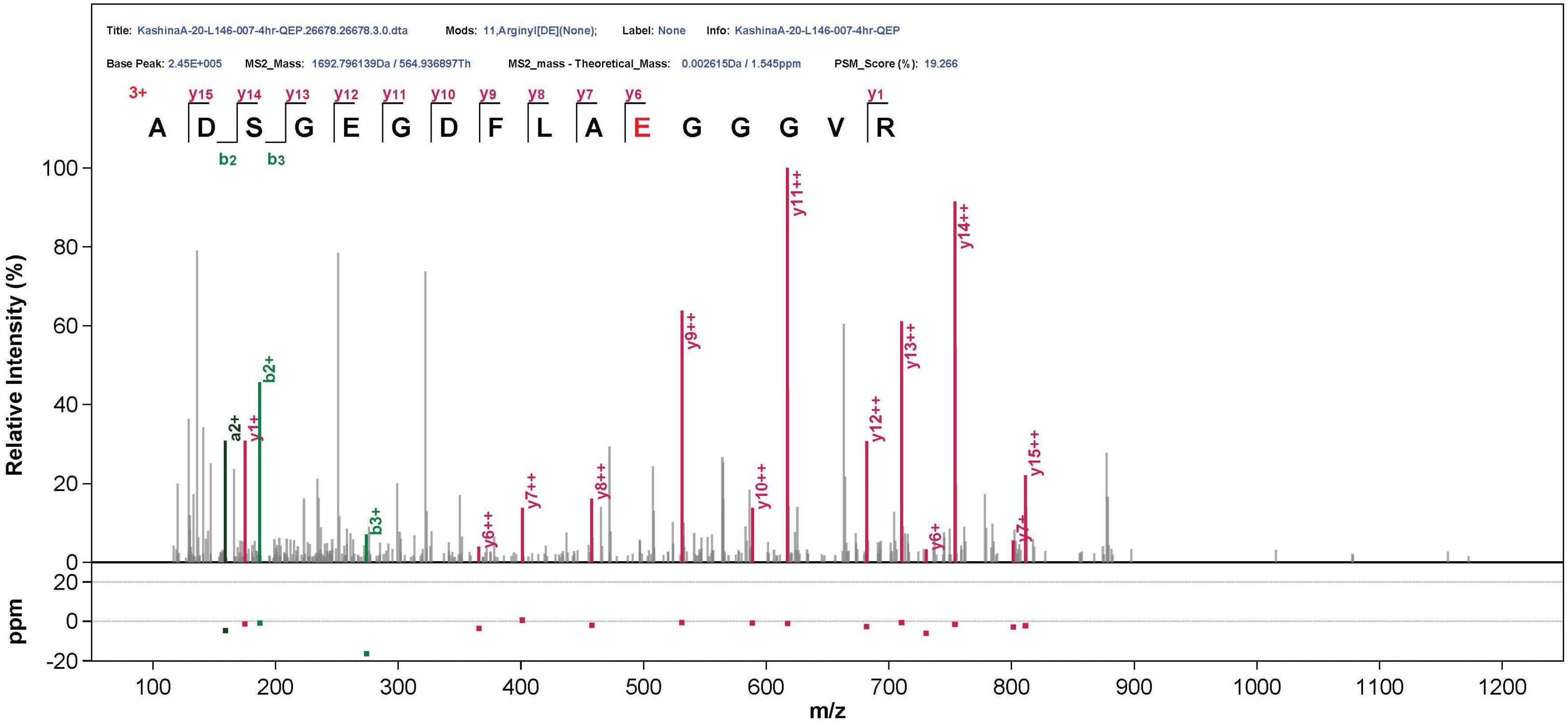

Apolipoprotein A4; E21

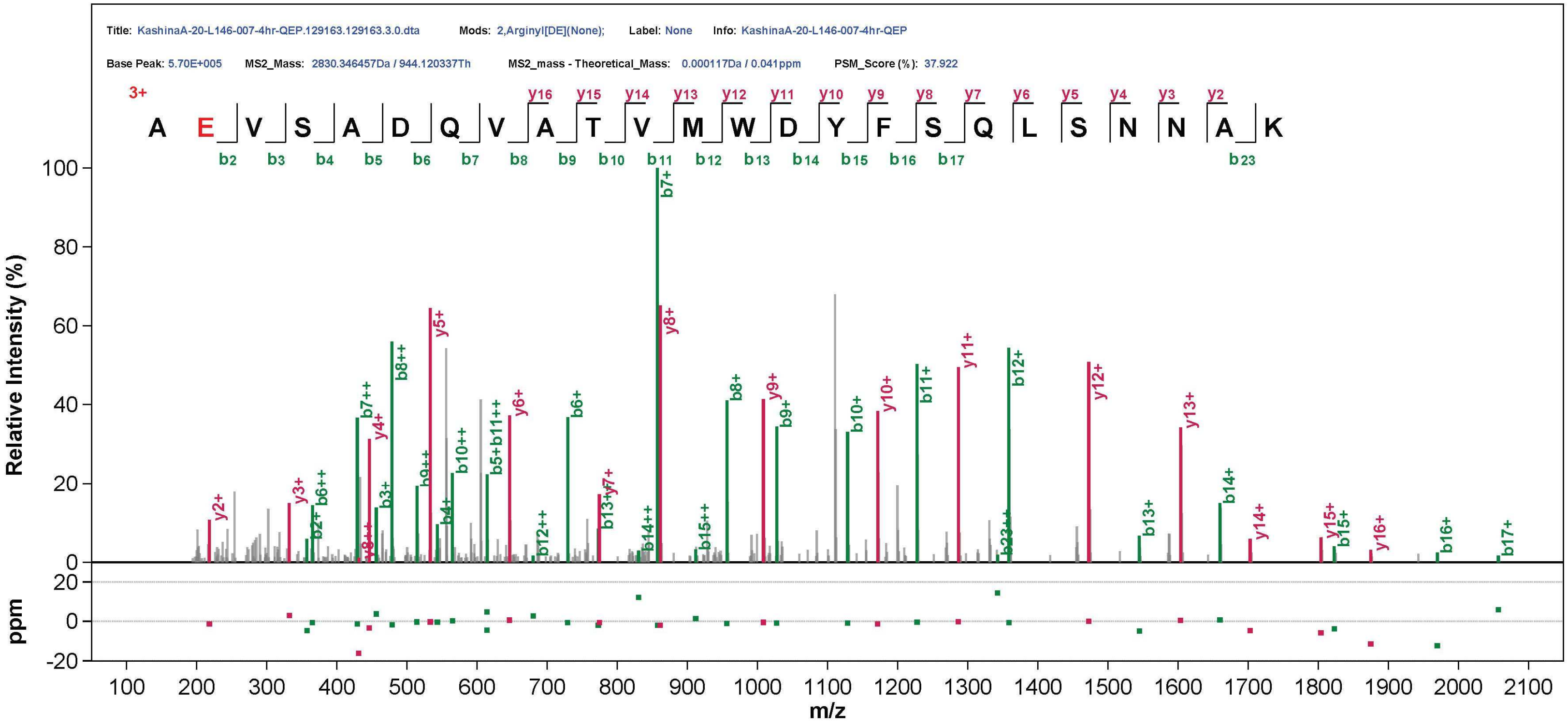

Antithrombin-III; E303

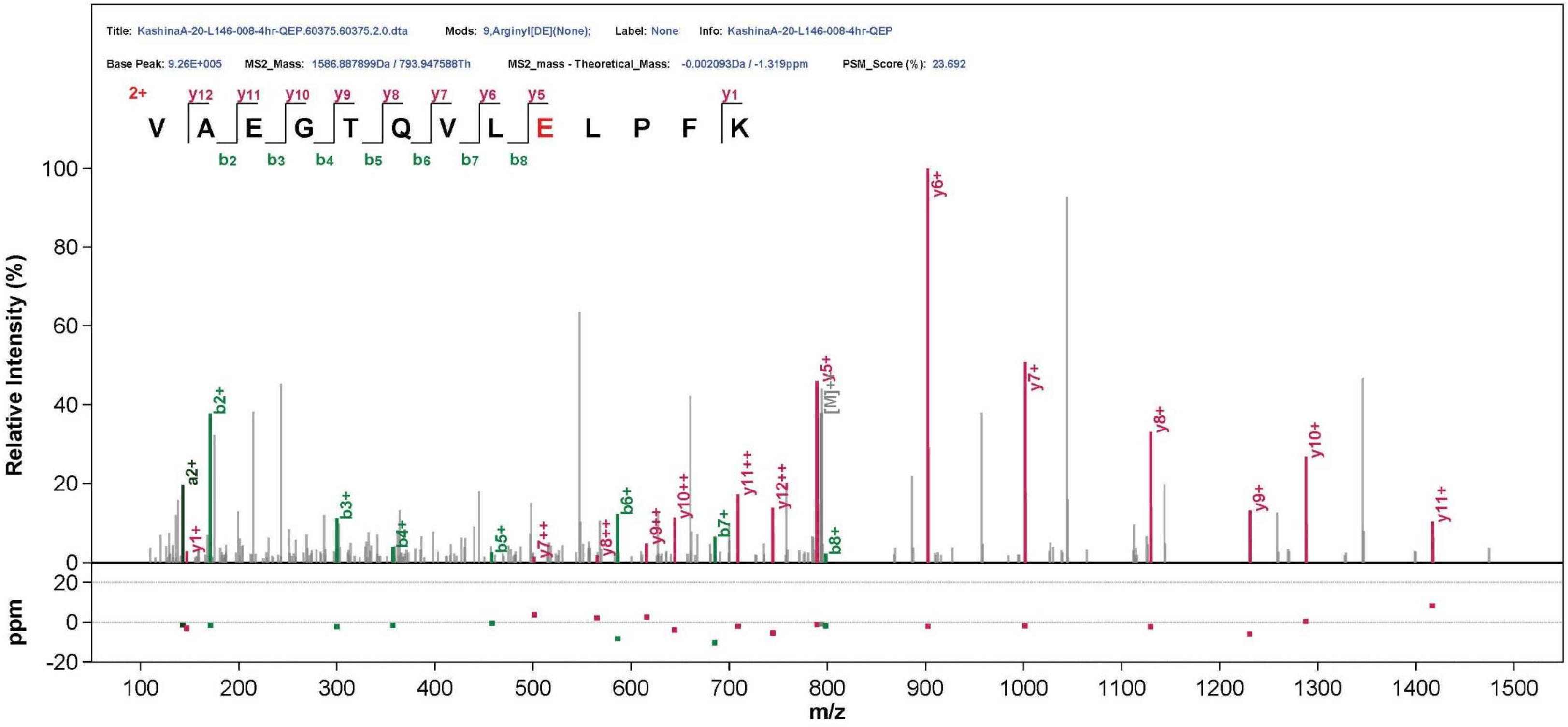

Transthyretin; E71

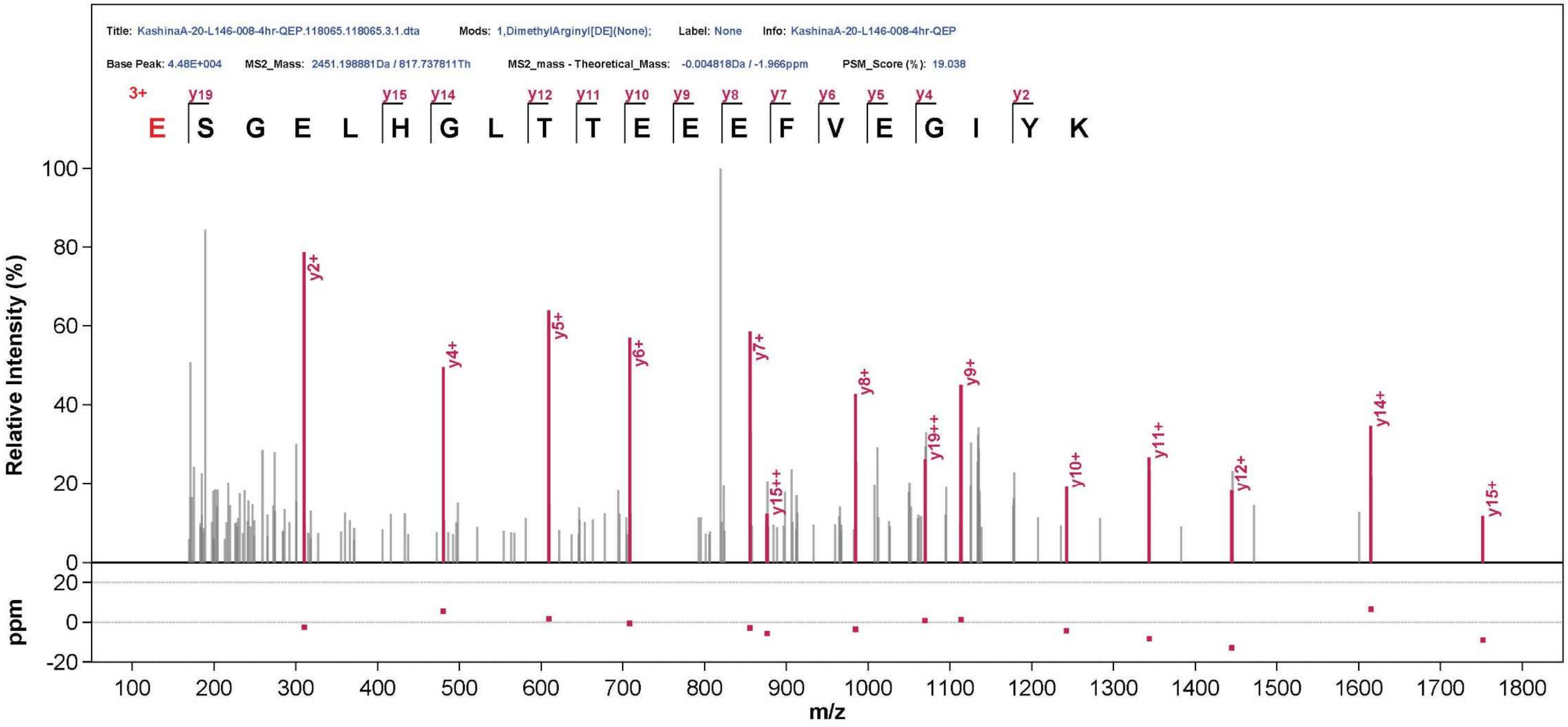

Antithrombin-III; E321

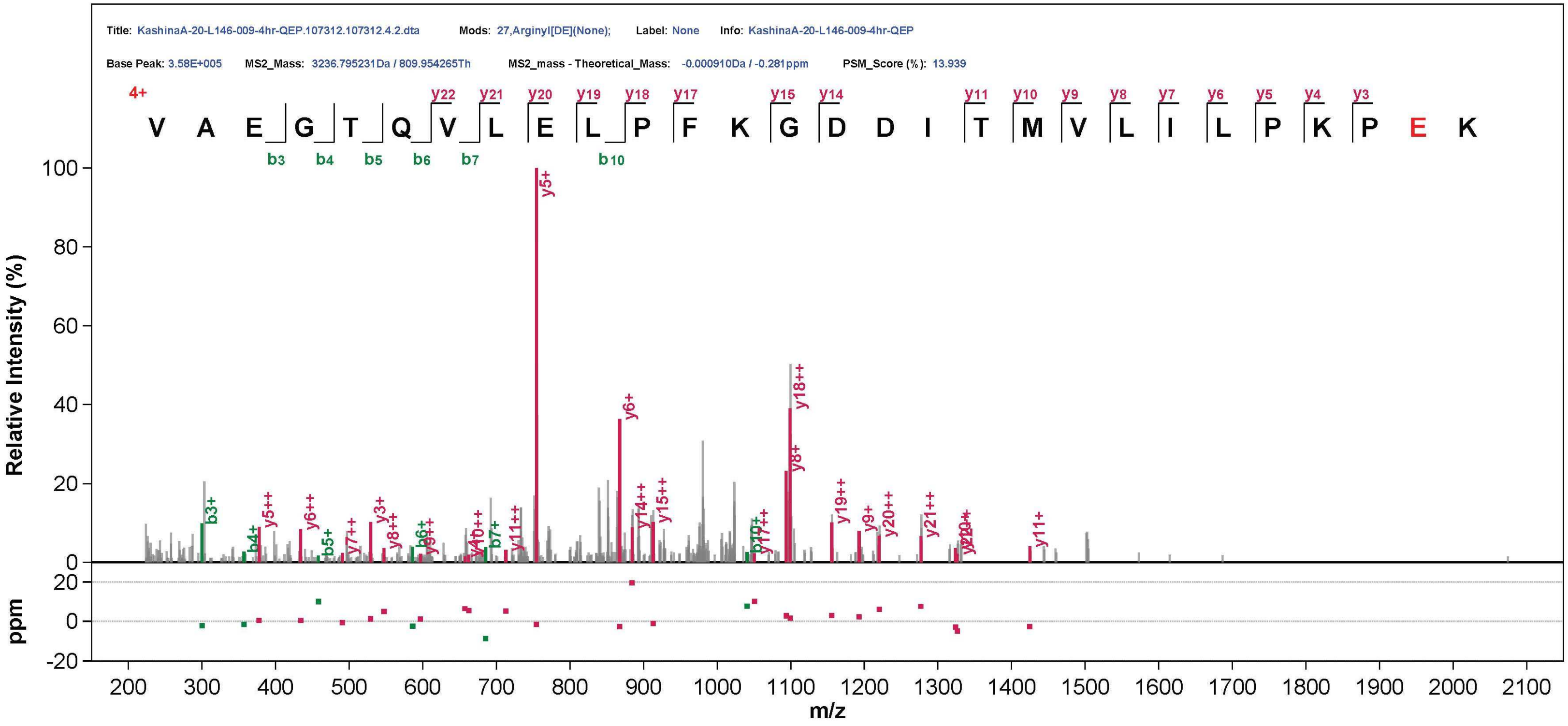

Supplement: Supplementary file 8 [file DataSheet1.PDF]
